# Case report of a giant cell tumor of the carotid sheath

**DOI:** 10.1016/j.radcr.2023.04.043

**Published:** 2023-05-23

**Authors:** Junjian Huang, Jason G. Newman, Bryan A. Pukenas, Apoorva Kotha, Michael Husson, Linda J. Bagley

**Affiliations:** a Department of Radiology, University of Pennsylvania Health System, 800 Spruce St, Philadelphia, PA 19107, USA; b Department of Radiology, University of Alabama at Birmingham, 1802 6th Ave St, Birmingham, AL 35233, USA

**Keywords:** Carotid sheath tumor, Giant cell tumor

## Abstract

Giant cell tumor of the soft tissue (GCTST) is a neoplasm with low malignant potential and typically affects the trunk and extremities. Herein, we present a case of a palpable right neck mass diagnosed as a GCTST of the carotid sheath in a 38-year-old woman. A review of the imaging characteristics as well as of the differential diagnoses of primary neoplasms of the carotid space is presented.

## Introduction

Giant cell tumor of the soft tissue (GCTST) is an extremely rare tumor that is considered as either a giant cell variant of malignant sarcoma or a soft tissue equivalent of a giant cell tumor of bone. This sort of tumors are typically found in the extremities with the femoral bone being the most common location (80%-90%) [1,2]. Although they are considered to be benign, there have been reports of metastasis and local recurrence in the literature [1]. Herein, we present a case of giant cell tumor of soft tissue in the neck region that originated from the carotid sheath treated with embolization.

## Case report

**Fig. 1:**
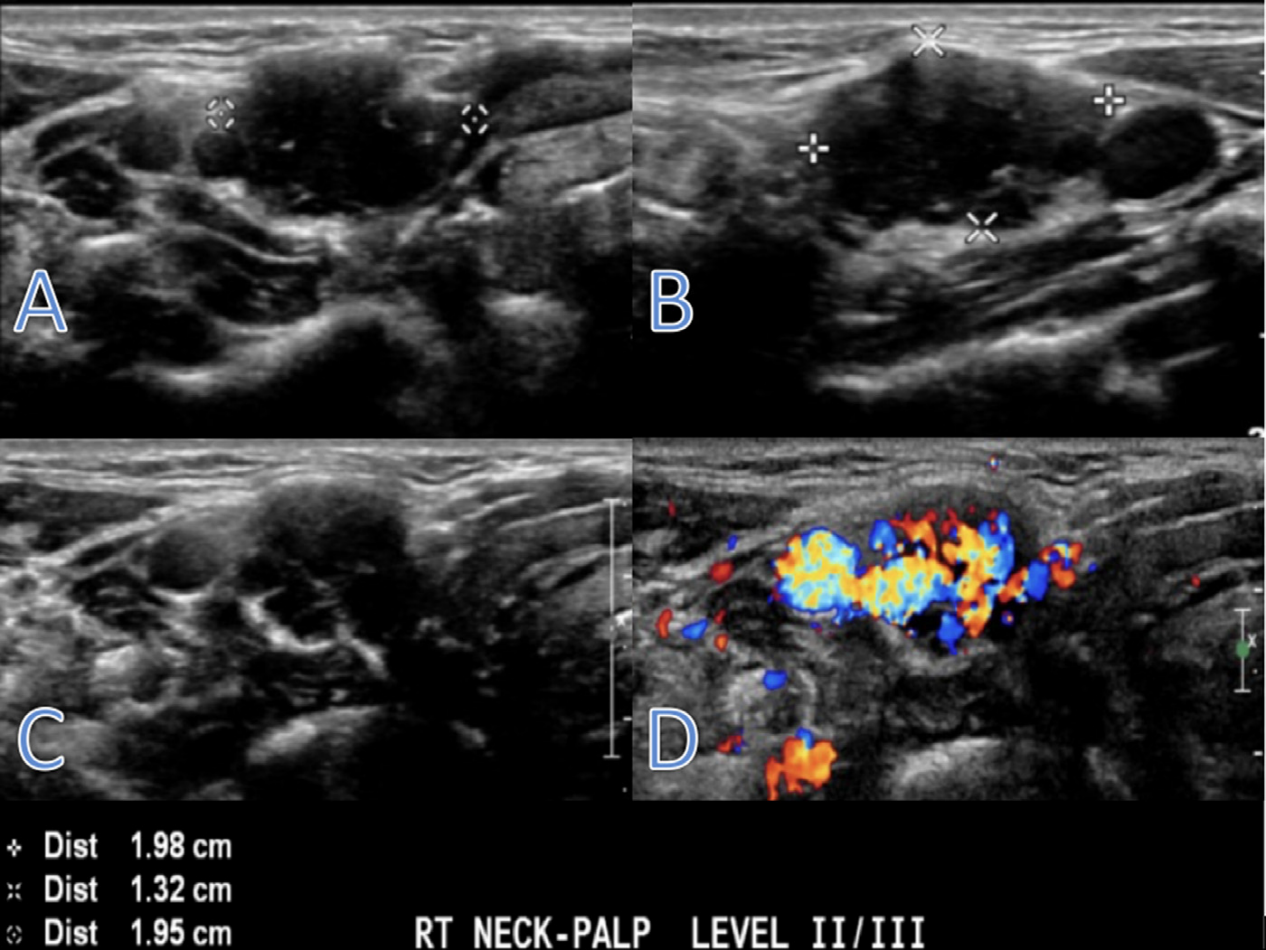
Ultrasound of the neck demonstrating hypoechoic vascular mass lesion just anterior to carotid bifurcation.

**Fig. 2:**
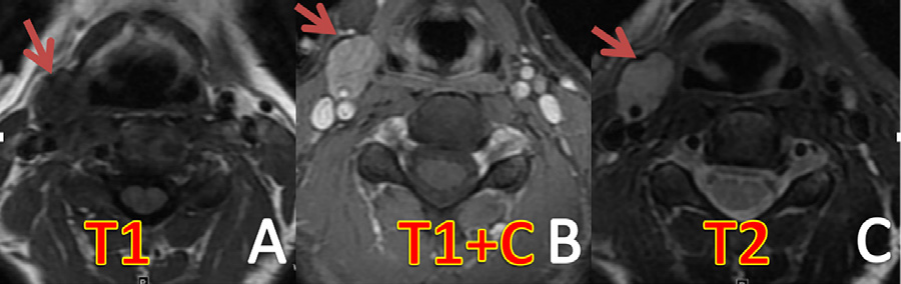
Axial T1-weighted (A), gadolinium-enhanced axial T1-weighted (B), and axial T2-weighted (C) MR images of the neck demonstrating homogenously enhancing approximately 2 cm minimally lobulated T2 hyperintense mass anterior to right common carotid artery bifurcation, contacting and likely encasing, but not significantly narrowing the proximal right external artery.

**Fig. 3:**
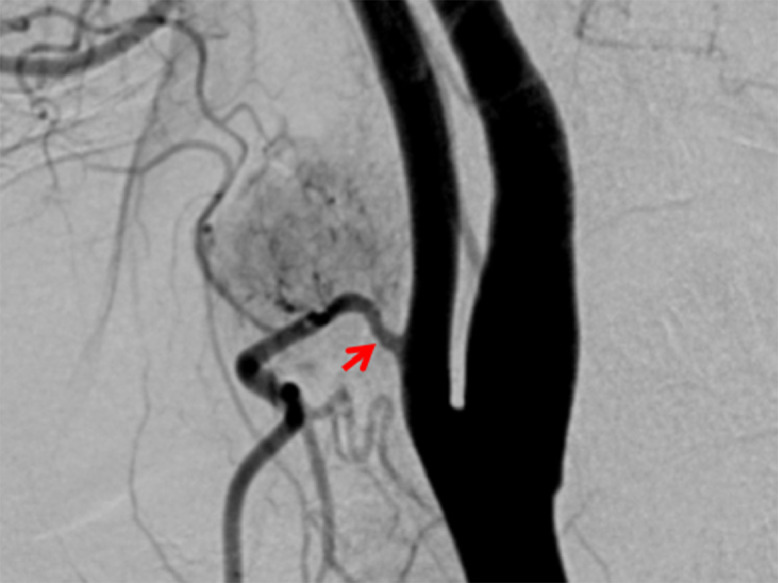
Digital subtraction angiography was performed with lateral view of the cervical vasculature, obtained during injection of the left common carotid artery demonstrated a rounded, moderately vascular mass lesion anterior medial to the right common carotid artery bifurcation, mildly displacing the right external carotid artery posteriorly with supply via multiple branches of the superior thyroidal artery. Note that the common carotid artery bifurcation is not splayed.

A 38-year-old woman presented with a painless right-sided neck mass (Table 1). The patient had no relevant medical history and physical examination revealed a palpable 1 cm firm, semi-mobile, and pulsatile mass in the right neck. Ultrasound (US) of the neck showed a hypoechoic, vascular solid nodule measuring 2 × 1.3 × 2 cm in size at level II/III of the right neck (Fig. 1). Magnetic resonance imaging (MRI) of the neck revealed a well-circumscribed, homogenous, T1 hypointense, T2 hyperintense, enhancing mass anterior to the carotid bifurcation, which contacted and possibly encased the right external carotid artery just beyond its origin (Fig. 2). Although some atypical features were present, a preliminary diagnosis of paraganglioma was considered. Consequently, the patient underwent angiography and embolization of the vascular mass (Fig. 3).

**Table 1:** Summary table.

Etiology	Unknown
Incidence	71 prior reported cases
Gender ratio	Female > male
Age predilection	30-50
Risk factors	Unknown
Treatment	Surgical resection
Prognosis	<25% chance of local recurrence.
Findings on imaging	T1 hypointense, T2 hyperintense, +gadolinium enhancement

**Fig. 4:**
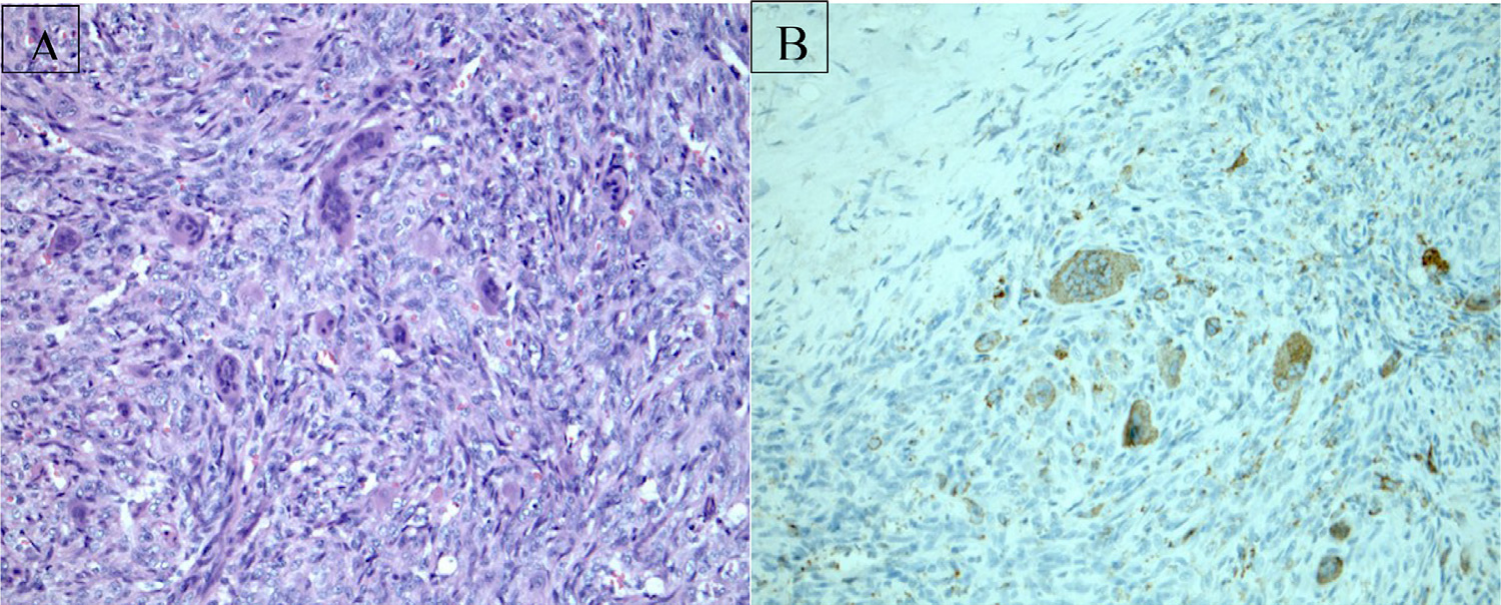
H & E stain showing immunostains positive for CD68 in the giant cells supporting diagnosis of giant cell tumor of soft tissue (A). CD68 immunohistochemical stain (B).

Surgical excision of the mass was performed, and the tumor demonstrated a nodular spindle cell proliferation with scattered giant cells (Fig. 4A). There was no significant atypia. Immunostains were positive for CD68 in the giant cells (Fig. 4B). These findings supported a diagnosis of giant cell tumor of soft tissue. The patient suffered no ill effects from the procedure and is recurrence-free 30 months following excision (Fig. 3).

## Discussion

GCTST of the carotid sheath is scarce, this is only the second case reported in literature of this tumor in the carotid sheath. These tumors are rare and composed of multinucleated giant cells and initially, they were thought to be identical to their bony counterparts; however, recent genetic analysis of the Gly34 codon of the H3F3A gene suggests that they may be pathogenically distinct [3], [4], [5], [6], [7]. To date, approximately 71 cases of giant cell tumor of soft tissue have been reported, and they have been shown to exist on a spectrum from benign to malignant, with local recurrence being reported in between 6.2%-25% of cases [8], [9], [10], [11], [12].

Primary neoplasms of the carotid sheath typically have a narrow range of differential diagnoses that include carotid body tumors (CBTs), glomus vagale, nerve sheath tumors such as schwannomas and neurofibromas, as well as metastatic lymphadenopathy and lymphoma (Table 2).

**Table 2:** Differential table.

DDX	MRI: Findings	Pattern of enhancement
Carotid aneurysm	Heterogeneous signal on T1- and T2-weighted imaging with or without blooming artifact on gradient echo sequences.	Avid enhancement
IJ or carotid thrombosis	Lack of flow void within the vessel.	No enhancement
Carotid dissection	Appearance varies based on chronicity. If the dissection acute the vessel may demonstrate a T1 hyperintense rim around the lumen due to intramural hematoma. May also so stages of thrombosis with laminated appearance.	Avid enhancement of the true lumen.
Carotid body tumor	T1 isointense, T2 hyperintense, salt & pepper appearance. Splaying of the ECA-ICA.	Rapid dynamic enhancement
Glomus vagale	Can be similar in appearance to carotid body tumors but salt and pepper appearance is less characteristic. Typically occur just below the skull base. DOES NOT splay the ECA-ICA.	Rapid dynamic enhancement
Schwannoma	Mild T1 hyperintense with T2 hyperintense rim. Target sign and fascicular sign can be seen. Nearly indistinguishable from neurofibroma.	Diffuse enhancement is typical.
Neurofibroma	Nearly indistinguishable from schwannoma. Target sign is seen more frequently in neurofibromas.	Diffuse enhancement
Giant cell tumor	T1 hypointense and T2 hyperintense signal	Homogenous enhancement

CBTs, paragangliomas located at the carotid bifurcation, can present as slow-growing, pulsatile masses and may involve cranial nerves. They typically splay the internal and external carotid arteries, and catecholamine secretion, although rare, can lead to hypertensive urgency. CBTs are associated with multiple endocrine neoplasia and von Hippel-Lindau and are the most common head and neck paraganglioma [[13], [14]].

Glomus vagale, another possibility, refers to paragangliomas of the neck that occur along the vagus nerve and tend to arise more cephalad than CBTs. These tumors often present with vocal cord paralysis due to impairment of the recurrent laryngeal nerve. Glomus vagale is the least prevalent of the primary carotid sheath neoplasms.

Nerve sheath tumors, including schwannomas and neurofibromas, are slow-growing, usually benign tumors of peripheral nerve sheaths that can cause a paroxysmal cough that may be elicited by palpating the mass. Vagal schwannomas displace the internal jugular vein and the carotid artery. Schwannomas are typically T2-hyperintense and do not demonstrate the ``salt and pepper'' signal intensity pattern on MRI. Neurofibromas may be more heterogeneous, may demonstrate central T2 hypointense (target sign), and are more likely to be central and inseparable from the nerve. Last, malignant peripheral nerve sheath tumors are often larger, more ill-defined, and painful [15].

When considering metastatic lymphadenopathy and lymphoma as potential differential diagnosis, additional considerations should be taken into account. Metastatic disease can have varied signal intensity characteristics, while lymphoma will typically be T2-hypointense, diffusion restricting, and avidly enhancing.

Aneurysms (patent or thrombosed) and thrombosed veins may occur in this location, and signal intensity, enhancement, and flow characteristics would vary depending on the degree of thrombosis. Lesions would also be inseparable from the parent vascular structure.

## Conclusion

Giant cell tumor of the soft tissue can occur in the carotid sheath and should be considered as part of the differential particularly when imaging characteristics do not follow that of other, more common carotid sheath neoplasms.

## Author contributions

Junjian Huang – Initial manuscript preparation

Jason Newman – Performed excisional surgery

Bryan Pukenas – Performed embolization

Apoorva Kotha – manuscript preparation

Michael Husson – Performed pathology interpretation, manuscript preparation

Linda Jane Bagley – Performed imaging interpretation and initial manuscript preparation.

## Human and animal rights

Not applicable.

## Patient consent

Informed consent was obtained from the patient as part of care at the University of Pennsylvania Health System.
